# Supplementary material for: Mode-matching metasurfaces: coherent reconstruction and multiplexing of surface waves
Source: Sci Rep. 2015 May 21;5:10529. doi: 10.1038/srep10529 (PMC4440216; doi:10.1038/srep10529)
Supplement: Supporting Information — Supplementary Figures 1-6 [file srep10529-s1.pdf]

Mode-matching metasurfaces: coherent reconstruction and multiplexing of surface waves

Authors: Jiao Lin,[1†*] Qian Wang,[2,3,4 †] Guanghui Yuan,[4,5] Luping Du,[4] Shan Shan Kou,[1] Xiao-Cong Yuan[6*]

Affiliations:

[1]School of Physics, University of Melbourne, VIC 3010, Australia

[2]Optoelectronics Research Centre & Centre for Photonic Metamaterials, University of Southampton, Southampton, SO17 1BJ, UK

[3]Institute of Materials Research and Engineering, Singapore 117602, Singapore

[4]School of Electrical and Electronic Engineering, Nanyang Technological University, Singapore 639798, Singapore

[5]Centre for Disruptive Photonic Technologies, Nanyang Technological University, Singapore 637371, Singapore

[6]Institute of Micro & Nano Optics, Shenzhen University, Shenzhen, 518060, China

†These authors contributed equally to this work.

*Corresponding authors: jiao.lin@unimelb.edu.au or xcyuan@szu.edu.cn

Supplementary Materials:

Figures S1-S9

Supplementary Materials:

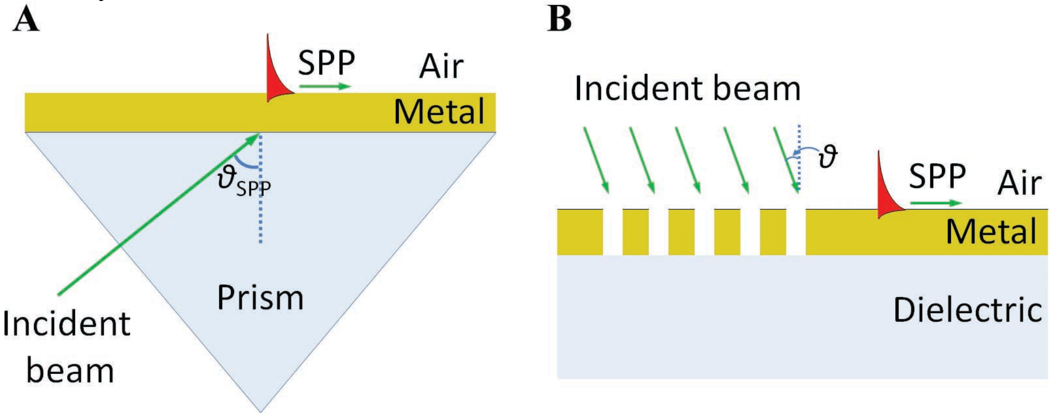

Fig. S1. Configurations for the excitation of surface plasmon polaritons (SPPs). (**A**) Excitation of SPPs at an air/metal interface with a prism. The thickness of the metal layer is typically at the range of tens nanometers in order to ensure the effective coupling of the electromagnetic field from one side to the other. (**B**) Excitation of SPPs with the help of a grating at the interface. The periodicity of the grating is designed to accommodate the incident angle of the light.

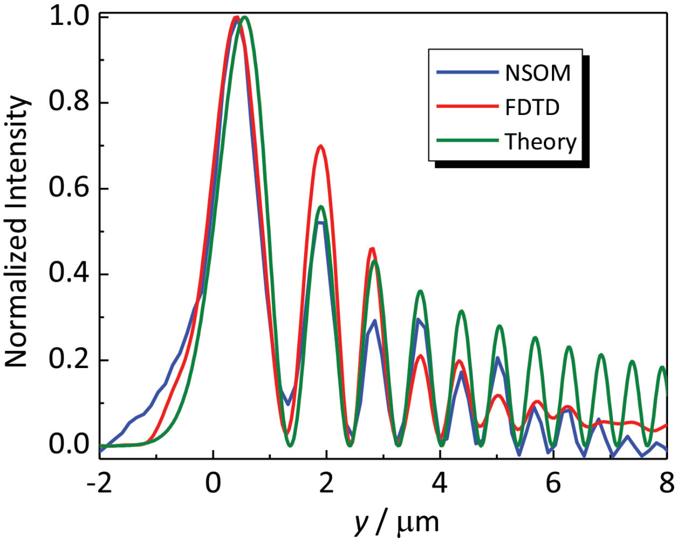

Fig. S2. The transverse intensity profile of the reconstructed PAB as compared to the theoretical prediction. The curves are obtained at the propagation distance $x = 2\mu m$ by near-field scanning optical microscopy in the experiment (in blue), finite-difference time-domain (FDTD) calculation (in red) and the theoretical expression in Eq. 2 (in green). The parameters of the PAB are $\lambda_{sp} = 613nm$, $a = 0.02$, and $y_0 = 600nm$. Although the binary design in Fig. 1C seems to give a uniform intensity distribution over the lobes along the y-direction, the real transverse distribution excited by the slits is in fact determined by a few more factors: the length of the slit and the finite transverse dimension of the metasurface. For instance, the length of the slit corresponding to the main lobe is much longer than those

corresponding to sidelobes (Figs. 1C&1D). This results in a much higher coupling efficiency from free-space light to SPPs for this slit. Therefore, the intensity at the main lobe in experiment is higher than the theoretical prediction. After normalized to the peak intensity at the main lobe, the transverse intensity profile appears to decay faster than the theoretical curve. The other reason for the faster decay is due to the finite transverse size of the metasurface. This effectively truncates the SPP wave, which is known to cause the nondiffracting wave to decay faster in the transverse dimension.

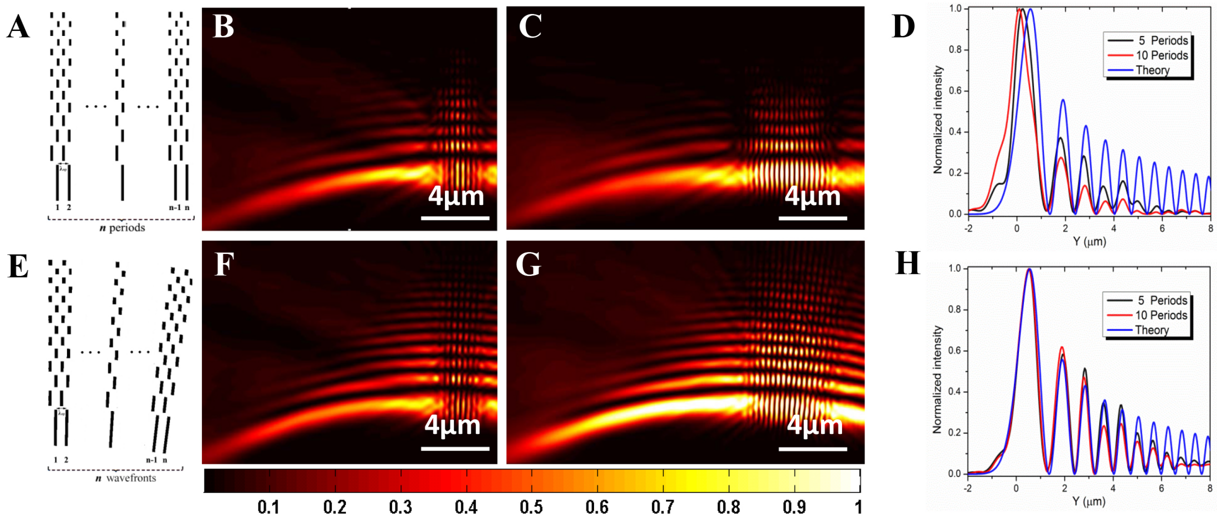

Fig. S3. Reconstruction of PABs by the use of plasmonic gratings with various number of periods. (**A**) A grating formed by repeating a single structure (obtained from the wavefront at a specific propagation distance) n times in the propagation direction. The pitch of the grating is equal to the wavelength λ_{sp} of the SPP. (**B**) & (**C**) The near-field intensity distribution of the reconstructed surface wave from design $\boldsymbol{A}$ when $n=5$ ($\boldsymbol{B}$) and $n=10$ ($\boldsymbol{C}$), respectively. (**D**) The corresponding transverse intensity profiles at $x=2\mu m$ for design $\boldsymbol{A}$. A more noticeable deviation from the theoretical curve is observed when the number of periods increases from 5 to 10. Ideally, a single structure ($n=1$) is sufficient to reconstruct the PAB though the efficiency would be poor due to the small cross-section as compared to the incident laser beam. However, a simple repetition of the structure does not offer the constructive interference among the individual PABs they generated. For instance, although the PABs generated by the first and last periods of the grating are identical, there is always a traverse dislocation between them because of the well-known parabolic propagation trajectory of PABs. Therefore, the SPP generated by a longer grating deviates more from the target PAB because of the larger difference among propagation distances of the constituent wavelets. For example, in this case, the wavelet emitted by the last period travels longer to catch up with the one emitted by the first period, which means a larger lateral offset between this two wavelet. (**E**) Design of an MPG matched to the field of a PAB covering n wavefronts. The structure is naturally curved as a result of the parabolic trajectory of the PAB. (**F**) & (**G**) The near-field intensity distribution of the reconstructed surface wave from design $\boldsymbol{E}$ when $n=5$ (**F**) and $n=10$ (**G**), respectively. (**H**) The

corresponding transverse intensity profiles at $x = 2\mu m$ for design ***E***. The surface waves reconstructed from the MPGs are in better agreement with the target PAB than by using design ***A***. And the increase of the size of the MPG (number of the wavefronts) shows a minimum effect on the fidelity of the reconstructed wave.

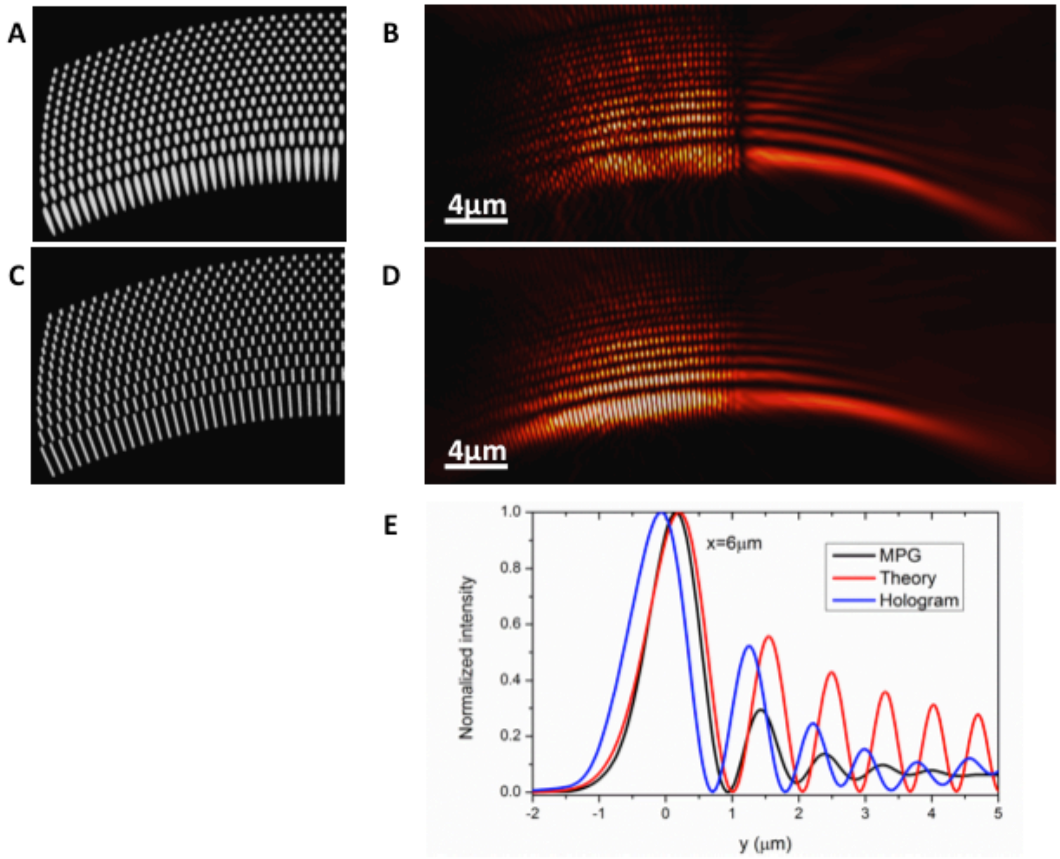

Figure S4. A surface hologram as compared with an MPG for the reconstruction of a PAB at normal incidence. (**A**) The holographic design is obtained by interfering the target PAB with a free-space planewave with the same frequency. The resultant 2D intensity distribution is then converted into a binary pattern as indicated in the figure. The parts in white indicating the places where the interference is constructive will be perforated through an Ag film. (**B**) The 2D intensity distribution (Ez) of the reconstructed SPP is observed in the full-wave calculation when the hologram is illuminated with a Gaussian beam (horizontally polarized) coming out of the plane. (**C**) An MPG designed for the reconstruction of the same PAB. (**D**) The reconstructed SPP obtained under the same condition as in ***B***. (**E**) The transverse intensity profiles (at the propagation distance $x = 6\mu m$) of the reconstructed SPPs (blue: hologram and black: MPG) and the target PAB (red). A good agreement between the SPP reconstructed from the MPG and the theoretical prediction of an ideal PAB (Eq. 2) is found whereas the SPP reconstructed from the hologram deviates from the theory. This can be attributed to the fact that the constituent aperture antennas (the parts in white) in the hologram vary in the width creating an uneven phase response across the device when illuminated with a plane wave. On the

contrary, the aperture antennas in the MPG have similar width and therefore free from the additional phase modulation giving a better reconstruction.

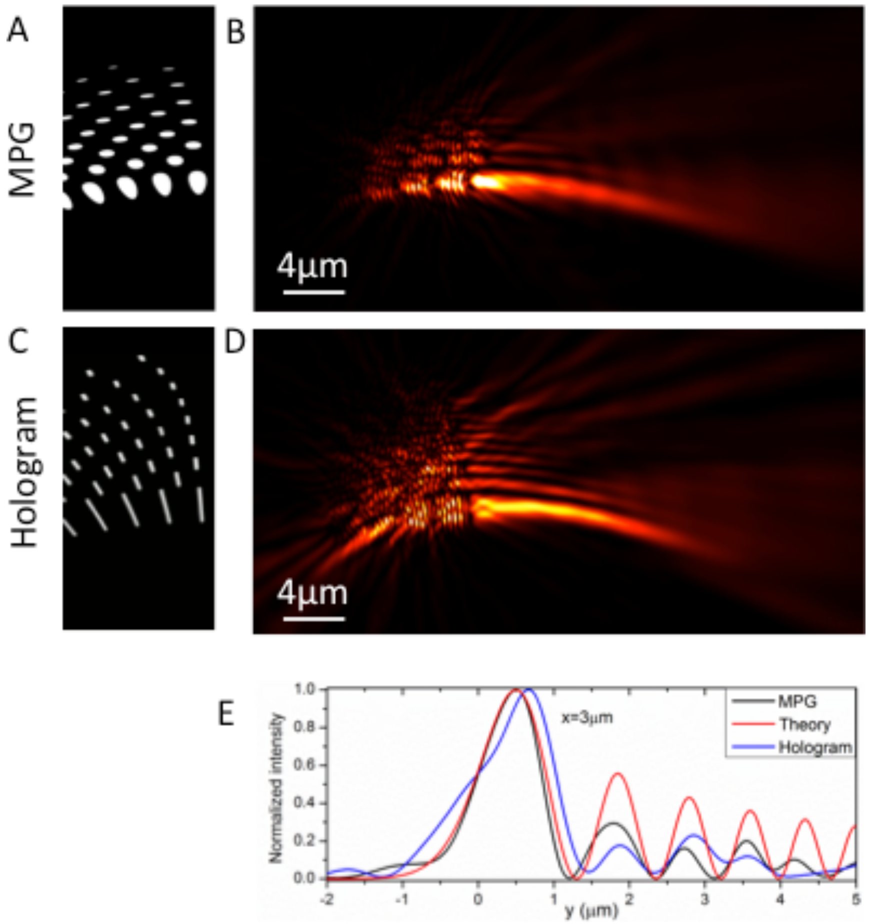

Figure S5. A surface hologram as compared with an MPG for the reconstruction of a PAB under an oblique illumination. (**A**) The holographic design is obtained by interfering the target PAB with a planewave tilted along the x-axis at 30º with respect to the normal of the surface. The resultant 2D intensity distribution is then converted into a binary pattern as indicated in the figure. (**B**) The 2D intensity distribution (Ez) of the reconstructed SPP is observed in the full-wave calculation when the hologram is illuminated by a Gaussian beam tilted at the same angle. (**C**) An MPG designed for the reconstruction of the same PAB. The MPG is obtained by subtracting the linear phase ramp ($kx\sin(\alpha)$ corresponding to the tilting angle α) from the phase distribution of the target PAB. (**D**) The reconstructed SPP obtained under the same condition as in ***B***. (**E**) The transverse intensity profiles (at the propagation distance $x = 3\mu m$) of the reconstructed SPPs (blue: hologram and black: MPG) and the target PAB (red). As compared with the holographic design, a better agreement between the SPP reconstructed from the MPG and the theoretical prediction of an ideal PAB (Eq. 2) is observed.

Comparison between MPGs and the holographic method

In principle, our method and the holographic method are both searching for the locations in the plane that are in phase. As the target surface wave is the same, we expect that all the appropriate methods (including MPGs and holograms) should produce very similar spatial distribution of scatterers. But the MPG is a more direct way without involving the interference with a reference wave. In addition, there are some subtle differences between the patterns obtained by the two methods:

(a) The shape of scatterers in the holographic design is oval (Fig. S6) as compared with the rectangular shape (Fig. S7) in the MPG. This is more evident in the case of oblique illumination.

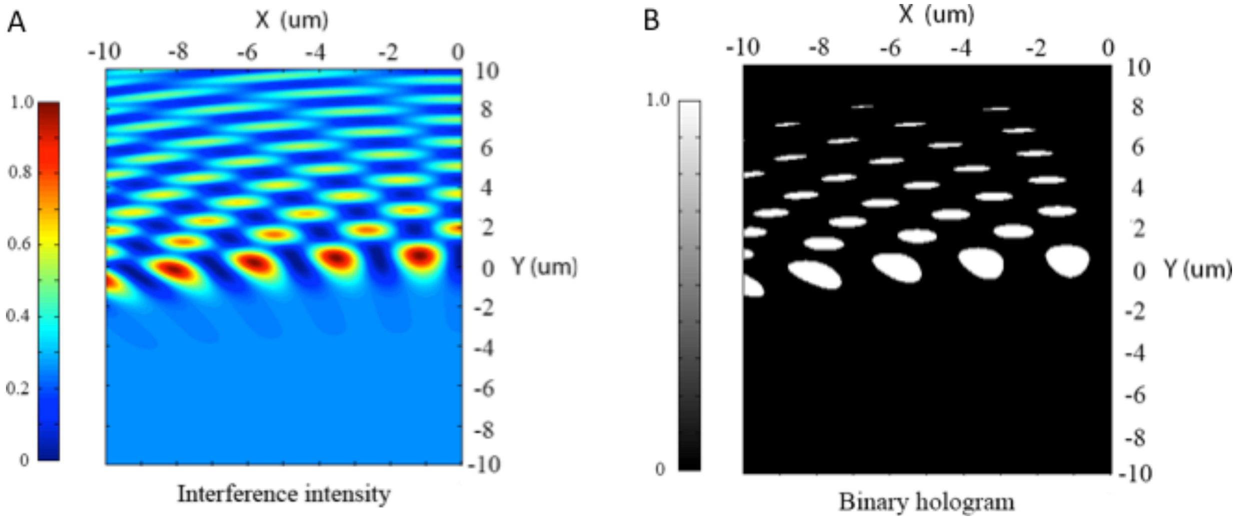

Figure S6. The holographic design for oblique illumination. (**A**) The intensity distribution obtained from the interference between the plasmonic Airy beam and a tilted plane wave; (**B**) The resultant binary hologram.

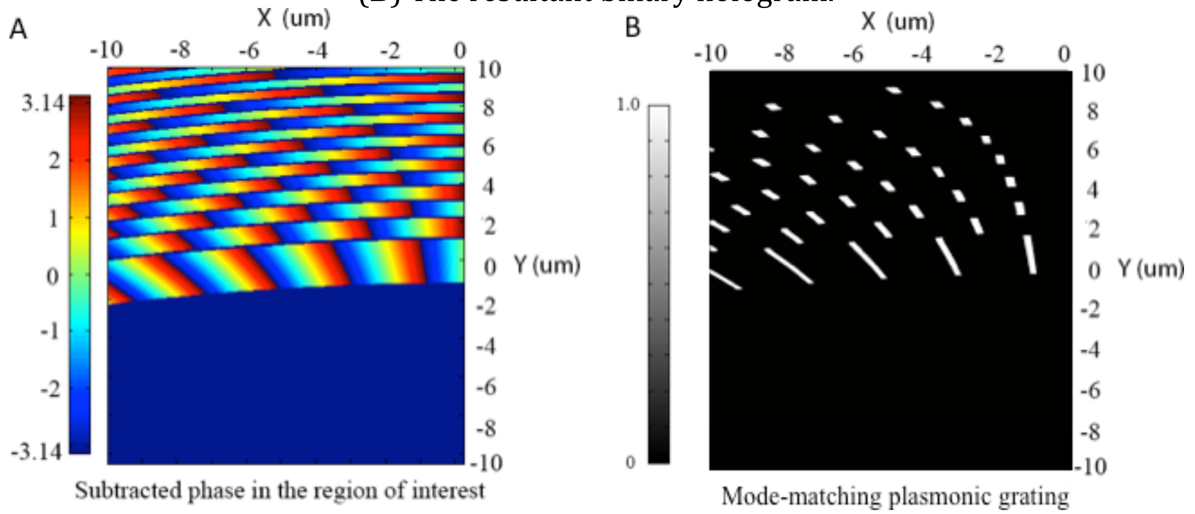

Figure S7. The design of an MPG for oblique illumination. (**A**) The compensated phase distribution in the region of interest (upper part of the image); (**B**) The resultant MPG.

(b) The pattern produced by the holographic method contains information of the amplitude distribution of the target surface wave, which modulates the size of the scatterers across the array. In general, the size of the scatterers is smaller at the places where the target surface wave has a lower intensity (e.g. sidelobes). This is very common in holograms and usually not an issue when scatterers are much larger than the wavelength of light. However, in our case, the dimensions of some of the scatterers produced by the holographic method are closer to the wavelength thus they act like small optical antennas. So the size of the scatterers will not only affect the local amplitude but also modulate the phase response of individual antennas. This adds to the complexity of the holographic design and needs additional steps to compensate the uneven phase response due to size change.

On the contrary, the MPG treats the phase and amplitude distributions of the target wave separately. The phase distribution is used to produce the slit antennas at the right positions and the width of the slit antennas remains as a constant. As a slit antenna is only responsive to the incident polarization state that is oriented along its short axis, the constant widths of slit antennas ensure that each of them produces similar phase responses. Then the amplitude distribution gives an area of interest where the amplitude of the target surface wave is above certain threshold. In the end, only the slit antennas fall into the area of interest will be kept (Fig 1A). The separate use of the phase and amplitude information of the target surface wave is much different from using the complex amplitude (phase and amplitude simultaneously) of the target wave in forming an interference pattern in the holographic method.

(c) The two patterns (Figs. S4A and S4B) resulted from the two methods are both binary, i.e. there are only two values in the patterns (1 for white, 0 for black). So it is difficult to reach the design in Fig. S4B from Fig. S4A by a simple binary image-processing step. In principle, it is still possible to convert Fig. S4A to Fig. S4B. However, it would be less preferred since getting Fig. S4B from the target surface wave is rather straightforward using mode-matching gratings.

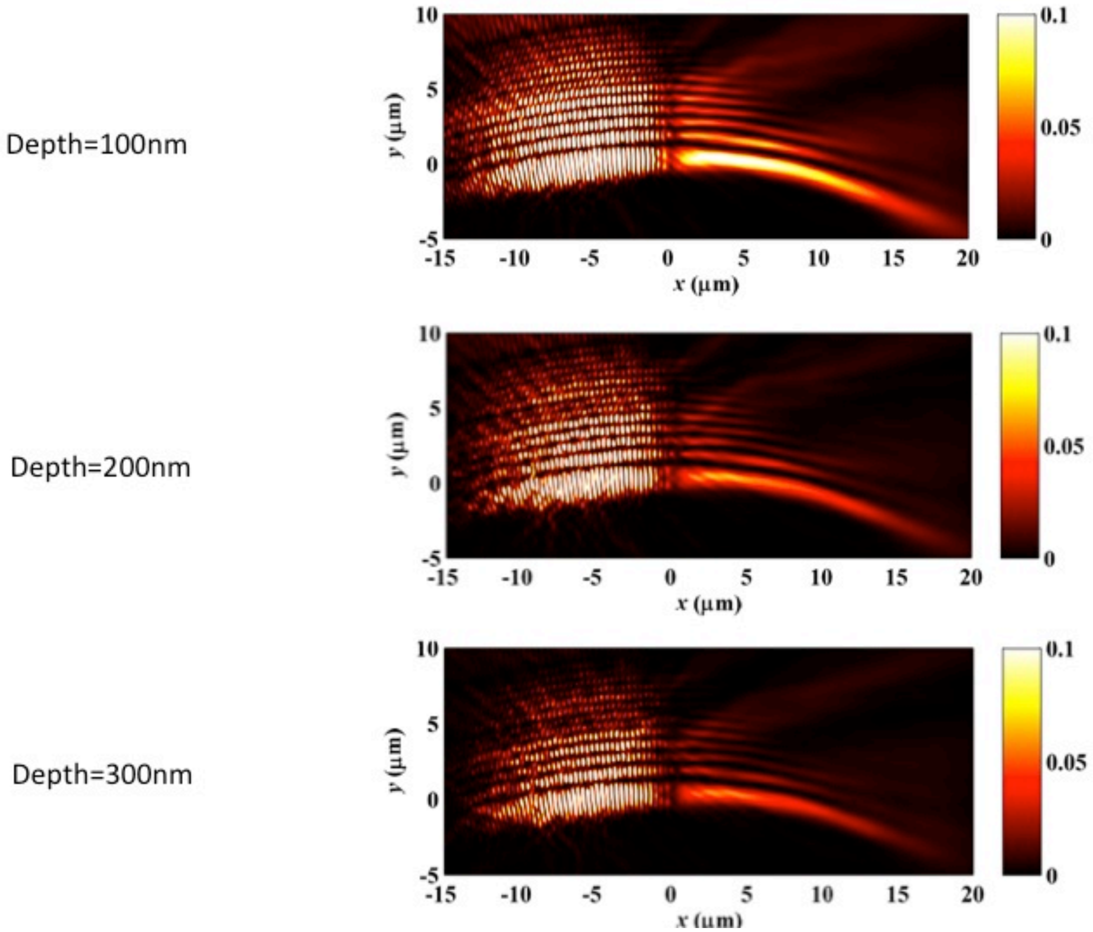

Figure S8. Near-field intensity distributions of SPPs generated by an MPG designed for the excitation wavelength of 633nm. The width of slits is 200nm.

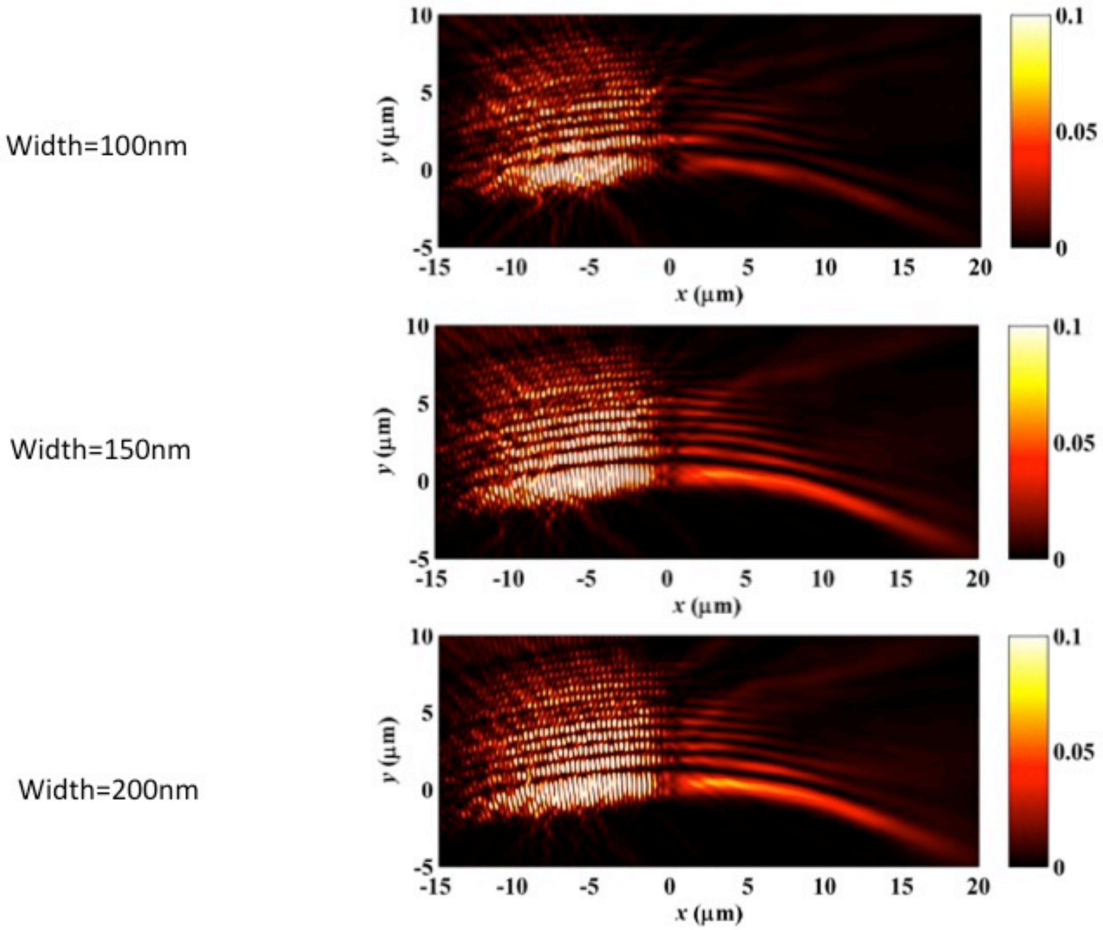

Figure S9. Near-field intensity distributions of SPPs generated by an MPG designed for the excitation wavelength of 633nm. The depth of slits is 200nm.
